# Preclinical efficacy of sepantronium bromide (YM155) in multiple myeloma is conferred by down regulation of Mcl-1

**DOI:** 10.18632/oncotarget.2529

**Published:** 2014-09-26

**Authors:** Verena Wagner, Dirk Hose, Anja Seckinger, Ludmila Weiz, Tobias Meißner, Thiery Rème, Iris Breitkreutz, Klaus Podar, Anthony D. Ho, Hartmut Goldschmidt, Alwin Krämer, Bernard Klein, Marc S. Raab

**Affiliations:** ^1^ Max-Eder Group Experimental Therapies for Hematologic Malignancies, German Cancer Research Center (DKFZ) and Dept. of Internal Medicine V, University of Heidelberg, Heidelberg, Germany; ^2^ Dept. of Internal Medicine V, University of Heidelberg, Heidelberg, Germany; ^3^ National Center of Tumor Diseases, University of Heidelberg, Heidelberg, Germany; ^4^ INSERM U1040, Montpellier, France; ^5^ Clinical Cooperation Unit Molecular Hematology/Oncology, German Cancer Research Center (DKFZ) and Dept. of Internal Medicine V, University of Heidelberg, Heidelberg, Germany; ^6^ CHU Montpellier, Institute of Research in Biotherapy, Montpellier, France

**Keywords:** apoptotic signaling, Mcl-1, multiple myeloma, survivin

## Abstract

The inhibitor-of-apoptosis family member survivin has been reported to inhibit apoptosis and regulate mitosis and cytokinesis. In multiple myeloma, survivin has been described to be involved in downstream sequelae of various therapeutic agents. We assessed 1093 samples from previously untreated patients, including two independent cohorts of 392 and 701 patients, respectively. Survivin expression was associated with cell proliferation, adverse prognostic markers, and inferior event-free and overall survival, supporting the evaluation of survivin as a therapeutic target in myeloma. The small molecule suppressant of survivin - YM155 - is in clinical development for the treatment of solid tumors. YM155 potently inhibited proliferation and induced apoptosis in primary myeloma cells and cell lines. Gene expression and protein profiling revealed the critical roles of IL6/STAT3-signaling and the unfolded protein response in the efficacy of YM155. Both pathways converged to down regulate anti-apoptotic Mcl-1 in myeloma cells. Conversely, growth inhibition and apoptotic cell death by YM155 was rescued by ectopic expression of Mcl-1 but not survivin, identifying Mcl-1 as the pivotal downstream target of YM155 in multiple myeloma. Mcl-1 expression was likewise associated with adverse prognostic markers, and inferior survival. Our results strongly support the clinical evaluation of YM155 in patients with multiple myeloma.

## INTRODUCTION

Multiple myeloma (MM) is a malignant disease that is characterized by clonal expansion of terminally differentiated B cells. Despite major advances in overall survival (OS) achieved by the introduction of novel agents within the last decade, MM remains an incurable malignancy in most cases [1].

Although very heterogeneous with respect to molecular characteristics, a common feature of MM cells is the relatively slow rate of cell proliferation in most newly diagnosed patients [2, 3]. Equally important for MM cell survival and therapeutic resistance are impaired mechanisms of apoptosis. The anti-apoptotic Bcl-2 family member Mcl-1, in particular, seems to be essential for MM cell survival [4-7].

Survivin is a member of the IAP (inhibitors of apoptosis) protein family, encoded by the *BIRC5* gene, and is highly expressed in cancer cells while virtually absent in most differentiated normal tissues [8, 9]. Furthermore, expression levels of survivin have been found to correlate with poor prognosis in colorectal, non-small-cell lung, prostate, and breast cancer, as well as in MM [10-14]. Functionally, survivin has been shown to counteract apoptosis induction upstream of effector caspases [15] and to have an essential role in cell proliferation by regulating spindle assembly and microtubule attachment to the kinetochore as a member of the chromosomal passenger complex [16, 17]. In MM, survivin has been implicated in the mechanisms of action of several therapeutic approaches. Specifically, inhibition of AKT, STAT-3 and NFkB signaling is associated with reduced intracellular gene expression and protein levels of survivin [18-20]. Long-term knockdown of survivin resulted in moderate inhibition of MM cell growth and increased drug sensitivity [21].

Although survivin appears to be an attractive therapeutic target from this initial set of data, it has never been analyzed in a large cohort of patients for its prognostic and therapeutic value. The portfolio of clinical grade survivin specific antagonists has been surprisingly limited [9]. Recently, a novel imidazolium-based small molecule suppressant of survivin, YM155, has been described. This compound was identified in a cell-based promoter activity assay and was described to specifically abrogate survivin gene (*BIRC5*) expression, resulting in preclinical activity in several tumor models both *in vitro* and *in vivo* [22, 23]. YM155 is currently being evaluated in phase II clinical trials in lymphoma, melanoma, as well as in cancers of the breast, lung, and prostate.

Based on these data we sought to re-assess the prognostic significance of *BIRC5* gene expression in CD138-purified MM cells from a cohort of 1093 previously untreated patients and to pre-clinically evaluate YM155 for its therapeutic potential in MM. *BIRC5* expression proved to be a powerful prognostic marker for event-free (EFS) and OS in two independent cohorts of patients. YM155 potently abrogates MM cell growth associated with inhibition of survivin expression. Furthermore, delineation of molecular sequelae in MM cells showed that down regulation of Mcl-1 appears to be an even more important downstream effector mechanism in MM cells exposed to YM155. Mcl-1 is expressed in all 1093 MM cell samples and is likewise associated with an adverse clinical prognosis.

Taken together, YM155 displays great therapeutic potential on MM cells via inhibition of survivin and, more importantly, Mcl-1 expression. Further clinical evaluation of this compound in MM is strongly warranted.

## RESULTS

### Aberrant expression of survivin gene transcripts in multiple myeloma

**Figure 1:**
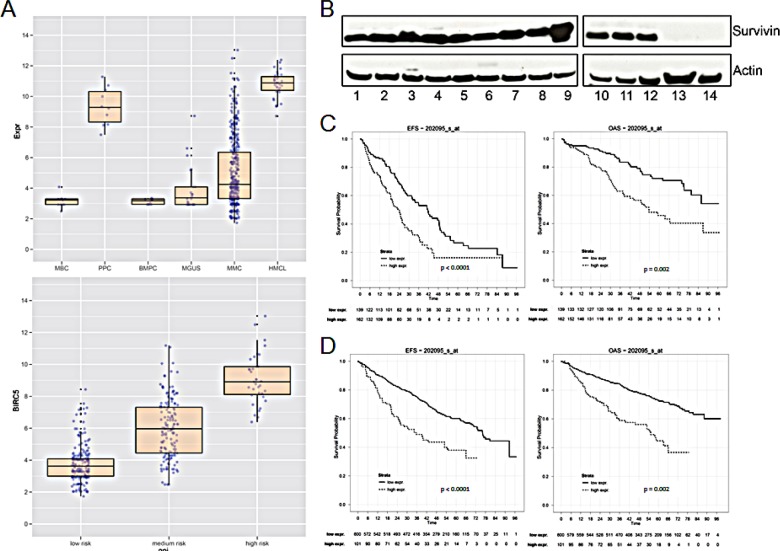
Survivin gene (BIRC5) expression and survival in multiple myeloma (A) Upper panel: logarithmic scale of BIRC5 expression in MBC (memory B-cells), PPC (polyclonal plasmablastic cells), BMPC (bone marrow plasma cells), MGUS, MMC (multiple myeloma cells) and HMCL (human myeloma cell lines). Lower panel: logarithmic scale of BIRC5 expression in patient samples with low, medium or high risk GPI. (B) Survivin protein expression in 12 different MM cell lines and in primary BMSC. The indicated samples were: 1 L-363; 2 KARPAS-620; 3 AMO-1; 4 OPM-2; 5 JJN-3; 6 EJM; 7 U-266; 8 RPMI-8226; 9 LP-1; 10 KMS-12-BM; 11 MOLP-8; 12 NCI-H929; 13 BMSC Patient1; 14 BMSC Patient2. (C) Event-free and overall survival in 301 patients undergoing high-dose chemotherapy at our center in Heidelberg with low or high BIRC5 expression, respectively. (D) UAMS Arkansas validation group: event-free and overall survival in 701 patients treated within the total therapy 2 and 3 protocols.

The survivin gene (*BIRC5, probe set 202095_s_at*) was not expressed in normal bone marrow plasma cells (n = 10, absent call 10/10), but was aberrantly expressed in 158/370 (42.7 %, present call) MM cell samples in our patient cohort. The mean expression in MM cells is 4-fold higher compared to that in normal plasma cells (P < 0.001) and 2-fold compared to plasma cells from patients with MGUS (p < 0.001), respectively (Figure 1A). In turn, *BIRC5* is expressed in all samples of proliferating normal or malignant plasma cells, *i.e.* polyclonal plasmablastic cells (PPC, non-malignant, n = 10), or human myeloma cell lines (n = 32) (Figure 1A). In both cases, the expression is significantly higher compared to MM cell samples (p < 0.001, p < 0.001). *BIRC5* expression in primary MM cells correlates significantly with proliferation as assessed by the gene expression profiling derived proliferation index, GPI (r = 0.81, p < 0.001) or propidium iodide staining [2] (r = 0.59, p < 0.001; n = 36), and the expression is significantly different between a low/median/high GPI (Figure 1A). *BIRC5* gene expression also increases with higher Durie-Salmon stage (p < 0.001, Suppl. Figure S1A). *BIRC5* expression was validated by qRT-PCR [14]. In agreement with microarray and qRT-PCR-data, survivin protein could be detected in 12/12 MM cell lines, being absent in 2/2 bone marrow stromal cell samples used as control (Figure 1B).

### Survivin expression, patient survival, and risk factors

In 301 patients undergoing high-dose chemotherapy, the presence of *BIRC5* expression was associated with inferior EFS and OS (22.6 vs. 35.4 months, p < 0.001; 52.9 vs. not reached, p = 0.002) using an optimized cutoff defined by maximum logrank statistics (Figure 1C). This strategy was also applied to 701 patients of the UAMS Arkansas group treated within the total therapy 2 or therapy 3 protocols with EFS of 12.3 vs. 54.1 months (P < 0.001) and OS of 17.4 vs. n.r. (p = 0.002) (Figure 1D). *BIRC5* expression is also significantly higher in patients harboring a del17p or gain of 1q21 (Suppl. Figure S1B). *BIRC5* expression is likewise higher in patients designated as “high risk” by the gene expression based risk scores of the UAMS (UAMS 70 gene score [24] or the IFM-score (IFM 15 gene score) [25] (Suppl. Figure S1C and S1D).

### YM155 specifically inhibits cell growth and induces apoptosis in myeloma cells

**Figure 2:**
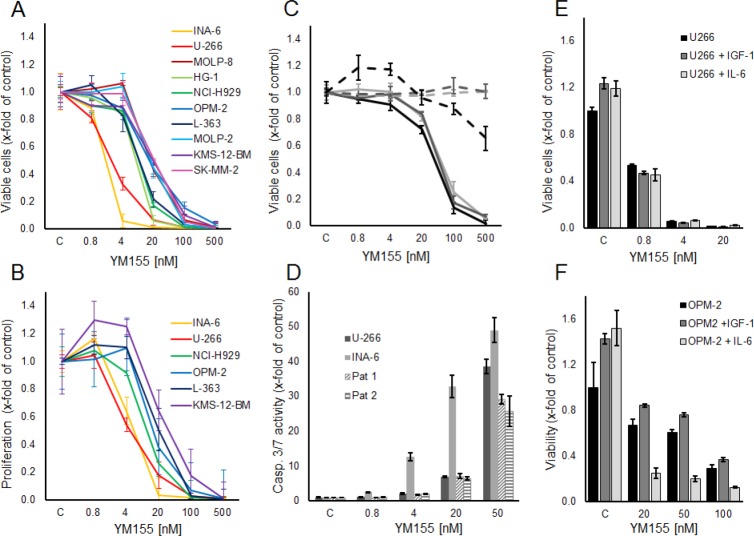
YM155 inhibits viability, proliferation and induces apoptosis in MM cell lines and primary MM cells (A) Viable and (B) proliferating myeloma cells are shown as percentage of control after 48 and 24 hours of treatment with YM155 at indicated concentrations or vehicle only, respectively. (C) Primary MM cells (solid lines) and BMSCs (dashed lines) from three patients. Viable cells as percentage of control after 48 hours of treatment with YM155. (D) Caspase 3/7 activity in U-266 and INA-6 cell lines and primary cells from two myeloma patients at 24 hours after treatment with YM155 at indicated concentrations. (E, F) IL-6 sensitizes OPM-2 cells to YM155. OPM-2 and U-266 cells were grown in starvation medium with or without IL-6 (50 ng/ml) or IGF-I (100 ng/ml), respectively, and YM155 at indicated concentrations. All quantitative data are shown as the mean +/− SD of 3 independent experiments conducted in triplicates.

The small molecule survivin suppressant YM155 has been shown to effectively reduce cellular survivin expression at the mRNA and protein levels [22]. In our panel of 10 MM cell lines, increasing concentrations of YM155 reduced cell growth (*i.e.,* viable cells after 48h) in all cell lines within a range of IC_50_ values of 2 nM to 50 nM (Figure 2A). Likewise, YM155 specifically inhibited proliferation in these cell lines already within 24 hours of exposure at similar IC_50_ values, assessed by [3H]-thymidine uptake (Figure 2B). Moreover, the viability of CD138-positive cells isolated from the bone marrow samples of three heavily pre-treated MM patients was also strongly reduced upon treatment with YM155, with only minor effects on corresponding primary bone marrow stromal cells, used as non-malignant controls (Figure 2C). In addition, YM155 induced apoptosis as detected by caspase 3/7 activation in the two most sensitive cell lines, U-266 and INA-6, as well as in primary myeloma cells, at concentrations comparable to those that inhibited proliferation (Figure 2D). Less sensitive cell lines, such as OPM-2, required a 10-fold higher concentration (500 nM) for induction of apoptosis (Suppl. Figure 2)

### IL-6 sensitizes OPM-2 cells to YM155

As the growth factors IL-6 and IGF-1 are known to promote MM cell growth and survival and to confer drug resistance, we next examined whether YM155 can overcome these protective effects. While both cytokines triggered MM cell growth in serum-free media in untreated controls, neither IL-6 nor IGF-1 were able to attenuate the growth inhibitory effect of YM155 in U-266 and OPM-2 cells (Figure 2E,F). Moreover, IL-6 strikingly sensitized OPM-2 cells to treatment with YM155 (Figure 2F). U-266 cells have been reported to utilize a constitutively activated IL-6/Stat-3 pathway by the autocrine secretion of IL-6 [27]. Considering that U-266 cells as well as the IL-6-dependent INA-6 cells were highly sensitive to YM155 under normal growth conditions, the fact that IL-6 stimulation in serum-free conditions sensitized OPM-2 cells to YM155 treatment suggested that YM155 might interfere with the IL-6/JAK/STAT3 signaling pathway.

### YM155 induces ER stress response signaling in myeloma cells

**Figure 3:**
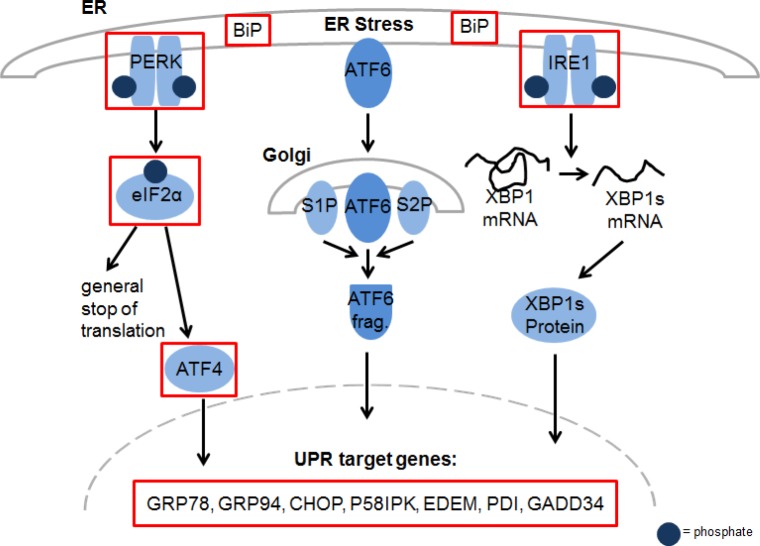
YM155 exposure regulates genes involved in ER stress in MM cells Activation of key components of the Unfolded Protein Response (UPR) in U-266 cells upon YM155 treatment. The marked genes were significantly upregulated (p < 0.05) after 12 hours of incubation at concentrations of 4 nM and 50 nM.

**Figure 4:**
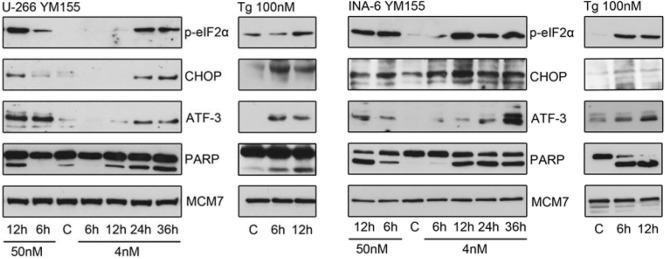
YM155 triggers ER stress signaling in MM cells UPR signaling pathways were assessed by immunoblot of p-eIF2α, CHOP and ATF-3 protein levels in U-266 and INA-6 cells at indicated times and concentrations. Cleaved PARP indicated onset of apoptosis, MCM7 served as loading control. thapsigargin (Tg) treated cells served as positive control samples.

To gain further insight into the mechanism of action of YM155 in MM cells, we performed serial gene expression profiling after 6 (4 nM and 50 nM), 12 (4 nM and 50 nM), and 36 hours (4 nM) of YM155 treatment in U-266 cells. Among significantly differentially regulated genes (P < 0.01), 189 were up-regulated and 63 down-regulated > 1.5-fold. Pathway analysis using the Ingenuity Systems software revealed up-regulation of genes associated with the endoplasmic reticulum (ER) stress/unfolded protein response (UPR) suggesting the involvement of this pathway in the anti-MM effects of YM155 (Figure 3). To confirm these data, we assessed key markers of the ER stress response on MM cells by immunoblot assay (Figure 4). PERK-dependent phosphorylation of eIF2a and induction of its downstream target CHOP together with the stress-related transcription factor ATF-3 were detected in U-266 cells within 24 hours at 4nM and within 6 to 12 hours (50 nM) of YM155 exposure. The up-regulation of CHOP, which has previously been shown to inhibit proliferation and to induce apoptosis in MM cells [29, 30], was closely associated with PARP cleavage, indicating the onset of apoptotic signaling. Similar results were obtained in INA-6 cells. Thapsigargin (TG) was used as a positive control for markers of ER stress response (Figure 4).

### YM155 inhibits IL-6/STAT3-signaling

**Figure 5:**
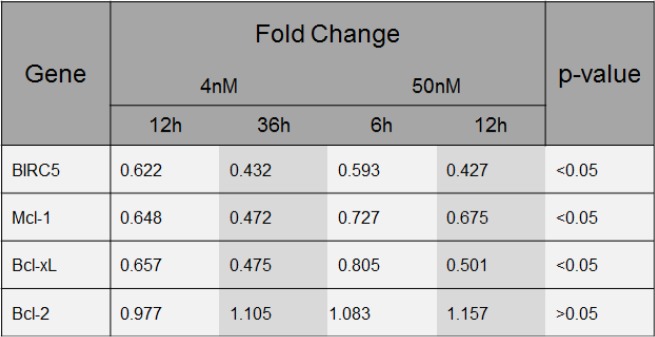
YM155 regulates gene expression of Bcl-2 family members Fold change of *BIRC5*, *MCL1*, *Bcl-xL* and *Bcl-2* gene expression in U-266 cells upon exposure to YM155 at indicated concentrations.

**Figure 6:**
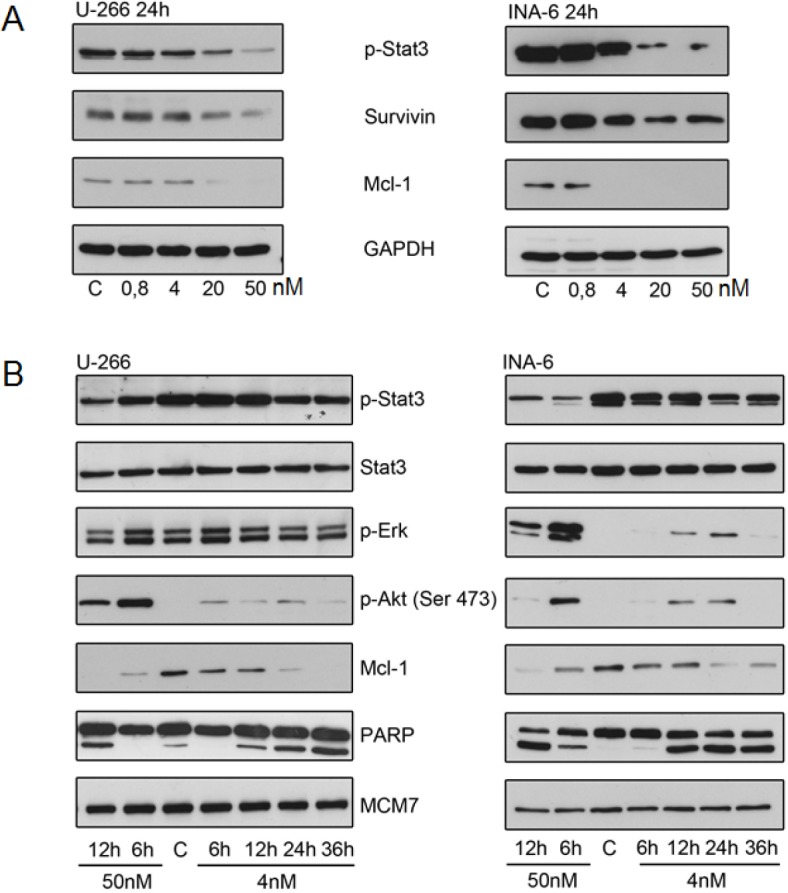
YM155 targets Mcl-1 in MM cells (A) p-Stat3, survivin and Mcl-1 protein levels after 24h of exposure to indicated concentrations of YM155. (B) Effect of YM155 on IL-6 signaling: p-Stat3, Stat3, p-Erk, p-Akt (Ser 473) and Mcl-1 protein levels assessed by immunoblot. Same lysates were used as in (Fig. 4); PARP and MCM7 blots are identical to (Fig. 4).

Gene expression profiling further suggested concordant down-regulation of genes that have been described to be regulated by the transcription factor STAT3 (Figure 5) [28]. While IL-6 signals via at least 3 pathways in multiple myeloma, namely STAT3, ERK1/2, and AKT [31, 32], we first biologically validated the impact of YM155 on IL-6/STAT3-signalling, finding a concentration-dependent decrease of phospho-STAT3 in both cell lines after 24 hours under normal growth conditions. This correlated closely with inhibition of survivin and Mcl-1 protein expression (Figure 6A). Phosphorylated STAT3 translocates from the cytoplasm to the nucleus. Immunofluorescence staining of STAT3 showed that nuclear STAT3 was depleted upon treatment with YM155 U-266 and INA-6 cells (Suppl. Figure 3).

There was also a strong time-dependent correlation between the inhibition of STAT3 activation and the decrease of Mcl-1. Apoptosis induction as assessed by PARP cleavage was observed to have a close temporal correlation with the reduction of STAT3 phosphorylation and Mcl-1 suppression (Figure 6B).

In contrast, we found a transient increase in the phosphorylation of AKT in both U-266 and INA-6 cells upon YM155 treatment, in particular at 50 nM of YM155. Similarly, phosphorylation of ERK was markedly induced in INA-6, while it was less pronounced in U-266 cells, indicating the activation of alternative signaling pathways (Figure 6B).

### YM155 targets Mcl-1 in MM cells

**Figure 7:**
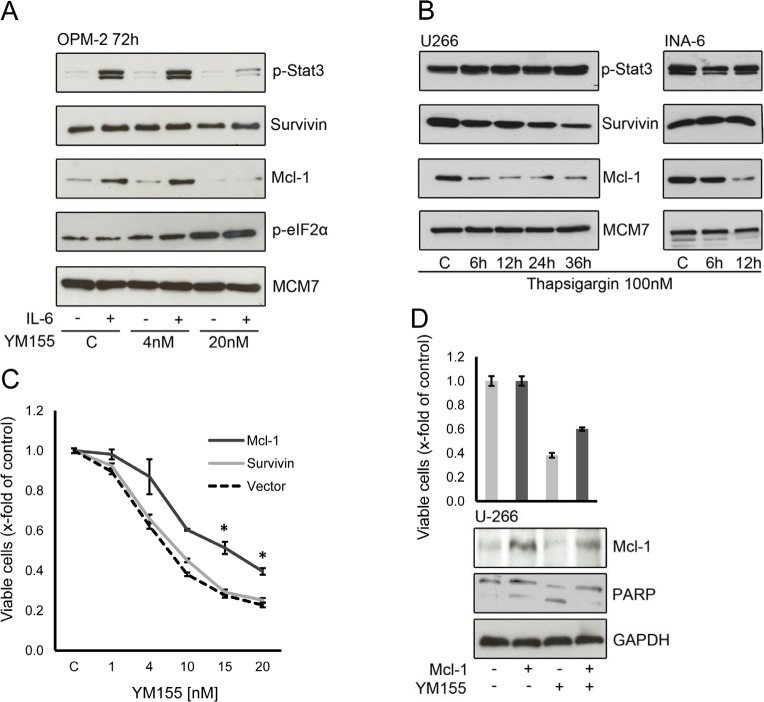
Mcl-1 is a critical target of YM155 in MM cells (A) Effect of YM155 on OPM-2 cells stimulated with IL-6, treated with YM155 at indicated concentrations or vehicle only and lysates were subject to immunoblot analyses of indicate targets. (B) Mcl-1 protein expression under ER stress induced by thapsigargin. Cells were lysed after 6 and 12 hours (U-266 and INA-6) and after 24 and 36 hours (U-266 only, as INA-6 cells were very sensitive to thapsigargin 100 nM). (C) Effect of ectopic Mcl-1 expression on cell viability. U-266 cells were transfected with a plasmid containing full-length Mcl-1 or with a vector control, respectively. After 24 hours YM155 was added at the indicated concentrations. Viable cells were measured 48h after transfection. The statistical significance of differences observed in Mcl-1 transfected vs. control cells was determined using an unpaired Student t test (*, p > 0.01). (D) Cell viability and corresponding protein levels of Mcl-1. U-266 cells were transfected with control or full-length Mcl-1 plasmid, respectively. YM155 was added at 10nM after 24 hours, cells were harvested 48h after transfection and subject to immunoblot analyses as indicated. PARP cleavage was used to asses onset of apoptosis, GAPDH served as loading control.

As IL-6 stimulation sensitized OPM-2 cells to treatment with YM155, we examined the phosphorylation of STAT3 and the protein expression of survivin and Mcl-1 in IL-6 stimulated cells exposed to YM155. IL-6 strongly induced STAT3 phosphorylation in OPM-2 cells (Figure 7A). YM155 inhibited this effect at a concentration of 20 nM in accordance to the sensitization to YM155 seen in the previous cell growth assays. Mcl-1 expression has been reported to be regulated by IL-6/STAT3 signaling [33] Here, Mcl-1 was markedly induced upon IL-6 stimulation and this effect was completely abrogated by treatment with 20nM of YM155. The constitutive expression of survivin in OPM-2 cells was not significantly enhanced by IL-6 and was not altered by exposure to YM155 in this concentration range (Figure 7A). This data suggests that down regulation of Mcl-1 rather than that of survivin might be the mechanism of action of YM155 in OPM-2 cells.

Survivin and Mcl-1 are proteins with a high turnover rate and are rapidly eliminated by proteasomal degradation [34, 35]. Activation of the ER stress response leads to a general inhibition of protein translation. We asked whether ER stress alone can lead to changes in survivin and Mcl-1 levels. We observed a rapid decrease of Mcl-1 in U-266 and INA-6 cells upon treatment with thapsigargin (Figure 7B). In contrast, only a moderate decline in survivin protein expression was seen in U-266 cells and no significant change in INA-6. Thapsigargin did not change phosphorylation of STAT3. This indicates that the activation of the ER stress response triggered by YM155 also contributes to the down regulation of Mcl-1.

YM155 induces alterations in two pathways – triggering the ER stress/UPR and selectively abrogating STAT3-dependent transcription factor activity – that seem to ultimately result in a depletion of the anti-apoptotic protein Mcl-1 in MM cells. We next sought to determine whether ectopic Mcl-1 expression is able to attenuate the anti-MM cell activity of YM155. As shown in figure 7C, exogenous Mcl-1 remarkably attenuates the inhibitory effect of YM155 on U-266 cell growth. Exogenous Mcl-1 protein levels were maintained in transfected cells and displayed reduced apoptotic cell death as assessed by PARP cleavage (Figure 7D). In contrast, exogenous expression of survivin had no significant impact on YM155 efficacy in MM cell growth (Figure 7C). This confirms that Mcl-1 depletion has a crucial role in the mechanism of action of YM155 in MM cells.

### *MCL1* gene expression and survival

Though ubiquitous, there is a consistently lower level of expression in MM cells and myeloma cell lines when compared to normal bone marrow plasma cells (Suppl. Figure S4A). Contrary to survivin, *MCL1* expression does not show an association with proliferation, but with adverse cytogenetic features (gain of 1q21 and presence of t(4;14)) and, at a statistically significant level despite a relatively small absolute difference, with gene expression based risk scores (Suppl. Figure S4B,4C and S4D).

In 301 patients undergoing high-dose chemotherapy, *MCL1* expression was associated with inferior EFS and OS (21.5 vs. 34.3 months, p = 0.005; 58.9 vs. n.r., p = 0.007) using an optimized cutoff defined by maximum logrank statistics. Similar results were obtained using data from 701 patients of the UAMS Arkansas group treated within the total therapy 2 and 3 protocols EFS of 65.1 vs. 79.2 months (p < 0.001) and OS of 86.4 vs. n.r. (p < 0.001) (Suppl. Figure S5A and S5B). This data confirm that elevated *MCL1* expression is associated with inferior EFS and OS in MM patients.

### YM155 in combination with other compounds

**Figure 8:**
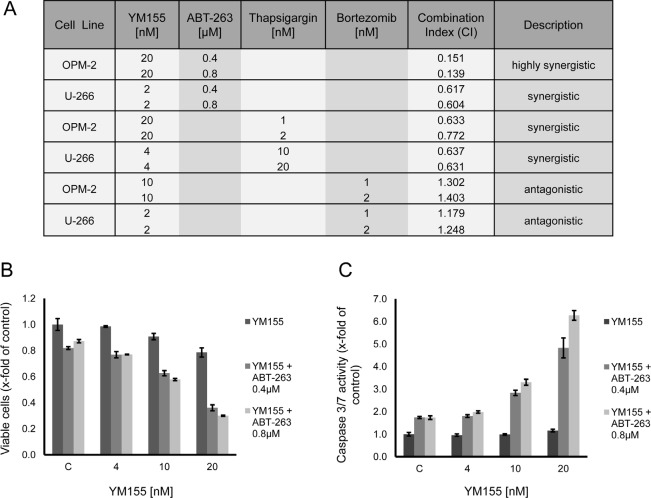
YM155 in combination treatments (A) Treatment of U-266 and OPM-2 cells with combinations of YM155 and ABT-263, thapsigargin or bortezomib. Viable cells were measured after 48 hours of treatment. Combination indices were calculated as described in material and methods. (B) Synergism of YM155 and ABT-263 on OPM-2 cells. Cells were treated with YM155 alone or in combination with ABT-263 (0.4μM or 0.8 μM, respectively). Viable cells were assessed after 48 hours. (C) Caspase 3/7 activity after 48 hours in OPM-2 cells treated with YM155 alone or in combination with ABT-263.

Bortezomib is known to trigger the ER stress response [36]. Low-dose YM155 (2nM and 10nM, respectively) was combined with low-doses of bortezomib (1nM and 2nM) in U-266 and OPM-2 cells. Isobologram analysis of cell growth demonstrated moderate antagonistic effects (CI 1.18 – 1.40) for this combination (Figure 8A). However, bortezomib requires the cleavage product of Mcl-1 to efficiently trigger apoptosis in MM cells [6] while YM155 abrogates Mcl-1 on a transcriptional level. In order to more specifically assess the potency of dual induction of ER stress, we combined YM155 (4nM and 20nM, respectively) with thapsigargin in both cell lines. As expected, moderately synergistic effects (CI 0.63 – 0.77) were seen with this combination (Figure 8A).

Recently, the co-dependency of MM cells on the anti-apoptotic Bcl-2 family members Mcl-1and Bcl-2/Bcl-xL has been reported [37]. Strikingly, co-treatment of OPM-2 cells with YM155 (20nM) and the antagonist of Bcl-2 proteins, ABT-263 (0.4 μM and 0.8 μM), led to a marked synergistic growth inhibition (CI 0.14 – 0.15) (Figure 8A) as well as to a highly synergistic activation of apoptotic signaling assessed by caspase 3/7 activity (Figure 8B and C). In contrast, the combination of these two compounds in U-266 cells was less effective but still synergistic (CI 0.60 – 0.61).

In summary, these results strongly support the early evaluation of YM155 in combination with inhibitors of other Bcl-2 family members in clinical trials.

## DISCUSSION

*BIRC5/SURVIVIN* is one of the most frequently over-expressed genes in all types of cancer [8]. The oncogenic activity of elevated survivin expression occurs through regulation of mitotic spindle assembly and cytokinesis, as well as through exerting anti-apoptotic effects by indirectly inhibiting caspase activation [38, 39]. In MM, *BIRC5* expression, detectable in 43% of myeloma cell samples, is associated with proliferation, prognostically adverse chromosomal aberrations (del17p and gain of 1q21), and gene expression based high-risk scores. *BIRC5* expression carries adverse prognostic significance regarding event-free and overall survival. It is thus a potential target for personalized and risk adapted treatment.

To explore survivin as a therapeutic target, we evaluated the cytotoxicity and mechanism of action of and some potential combination partners for the novel small molecule suppressant of survivin, YM155, now called sepantronium bromide. YM155 potently inhibits cell growth and induces apoptotic cell death in MM cells in a low nanomolar range, similar to what has been shown preclinically for prostate, colon, lung and a wide variety of other cancer entities [23]. These studies have already led to several phase II clinical trials with data now available on relapsed and refractory prostate cancer and diffuse large B-cell lymphoma [40, 41]. The excellent tolerability of the compound was demonstrated in these trials and single agent anti-tumor activity was reported. However, though there was one complete response in the lymphoma trial, overall relatively few patients responded. Ongoing clinical trials will therefore focus on drug combinations based on pre-clinically proven synergy.

We further aimed to dissect the molecular mechanism of action in MM cells. Although Cheng et al. describe YM155 as highly selective to suppress survivin promoter activity, the concomitant abrogation of Mcl-1 transcription was recently shown by Tang et al. in a panel of cell lines from solid tumors [42, 43]. While YM155 does suppress survivin levels in MM cells at higher concentrations, our data indicate that YM155 additionally triggers the ER stress response and abrogates both IL-6 induced as well as constitutive STAT3 signaling, ultimately converging on the inhibition of Mcl-1 expression, an anti-apoptotic factor that has been proven to be essential for MM cell survival [4, 44]. The potent anti-tumor activity of YM155, at least in MM, therefore seems to be mediated by the activation of the unfolded protein response and simultaneous abrogation of IL-6/STAT3 signaling. Mcl-1, rather than survivin, appears to play the major role in YM155-mediated growth inhibition and cell death in MM cells. This is further underlined by the reversal of YM-155 inhibition by forced expression of Mcl-1, whereas forced expression of survivin had no effect. In addition, knock-down of *BIRC5*/survivin by siRNA did not significantly inhibit growth and survival of MM cells (data not shown).

Based on these results, we re-evaluated the expression, association with molecular entities and prognostic significance of Mcl-1 in multiple myeloma. In contrast to survivin, *MCL1* expression does not show an association with proliferation, but with adverse cytogenetic features. In line with these findings, high *MCL1* expression is associated with adverse survival, confirming and extending data previously reported [45].

How does YM155 suppress expression of survivin and Mcl-1? Firstly, both Mcl-1 and survivin expression have been found to be regulated by STAT3 [33, 46, 47]. Specifically in MM, STAT3 dependent expression of Mcl-1 has been reported in INA-6 cells [48]. In addition, Mcl-1 and survivin, both proteins with short half-lives, are conceivably suppressed by the general inhibition of protein translation triggered by the unfolded protein response, as our data demonstrate that induction of ER stress alone leads to reduced protein levels of Mcl-1 and survivin. Furthermore, YM155 appears to disrupt the formation of a critical complex of transcription factors at the survivin core promoter by modulating subcellular localizations of Sp1 and ILF3/p54(nrb) [42, 49]. Interestingly, both Mcl-1 and survivin share transcriptional regulation by SP1, providing an additional molecular basis for the dual suppression of these critical pro-survival factors in cancer cells [50].

As the early first clinical trial data suggest that the single agent activity of YM155 is modest, appropriate combination partners based on preclinical synergy are likely to be required for the successful clinical application of this compound. Recently, a complex interaction pattern between anti-apoptotic Bcl-2 family members in MM cells has been reported [37]. This work describes a co-dependency of MM cells on Mcl-1 and Bcl-2/Bcl-xL. In line with these recent reports, our findings confirm a strikingly synergistic efficacy of the combination of YM155 with the antagonist of Bcl-2 proteins ABT-263, an orally active derivative of ABT-737 that selectively binds Bcl-2, Bcl-x_L_ and Bcl-w [51, 52]. We therefore propose to further evaluate YM155 in combination with inhibitors of the Bcl-2 family in future clinical trials.

Taken together, these results provide the theoretical framework for clinical trials targeting survivin and Mcl-1 in patients with MM.

## Materials and methods

### Materials

YM155 and ABT-263 were purchased from Selleck Chemicals (Houston, TX). Thapsigargin, Hydroxychoroquin, IL-6, and IGF-I were obtained from Sigma Aldrich (St Louis, MO). Primary antibodies were obtained from Cell Signaling Technology (Danvers, MA): Survivin, p-STAT3, STAT3, p-ERK, p-AKT (Ser 473), PARP, p-eIF2α, CHOP and from Santa Cruz Biotechnology (Santa Cruz, CA): Actin, Mcl-1, GAPDH, MCM7, ATF-3.

### Cell Culture

Human MM cell lines were purchased from the German Collection of Microorganisms and Cell Cultures (Braunschweig, Germany) and cultured as recommended. INA-6 cells, kindly provided by Dr. Renate Burger, University Hospital Schleswig Holstein Kiel (Kiel, Germany), were grown in RPMI-1640, 10% FCS (GIBCO/Invitrogen, Karlsruhe, Germany), with 2.5 ng/ml IL-6. The human MM cell line HG-1 was generated in the Multiple Myeloma Research Laboratory Heidelberg (unpublished data). All cell lines were periodically tested for contamination with mycoplasma by PCR and authenticated by DNA fingerprinting within our cell banking system according to the regulations of the German Cancer Research Center (DKFZ).

### Primary cells, human myeloma cell lines, Gene Expression Profiling, iFISH

Patients with previously untreated MM (n=370), individuals with monoclonal gammopathy of unknown significance (MGUS, n=22), and 10 healthy donors presenting at the University Hospitals of Heidelberg and Montpellier were included in the study after written informed consent was obtained in accordance with the Declaration of Helsinki, which was approved by the institutional review boards of the University Hospital of Montpellier, France and of the Medical Faculty of the Ruprecht-Karls-University Heidelberg (#229/2003 and S-152/2010), Germany. A total of 301 patients underwent front line high-dose conditioning chemotherapy with 200 mg/m² melphalan and autologous stem cell transplantation. Purification of normal bone marrow plasma cells, MM cells, plasmablasts and bone marrow stromal cells as well as gene expression profiling were performed as previously described [2, 53-55]. Interphase fluorescence in situ hybridization analyses were done on CD138-purified plasma cells as published [56]. CD27^+^ memory B cells (MBCs, n=11) were purified from buffy coats purchased from the French Blood Center (Toulouse, France) and plasmablasts (PPC, n=10) generated using an *in vitro* model described previously [57]. Thirty two human myeloma cell lines were fully characterized and microarray profiled as described [58]. Microarray data on cell lines are available in the ArrayExpress public database under accession numbers E-TABM-937 and E-TABM-1088.

### Statistical analysis

To assess for the presence or absence of gene expression, the “Presence-Absence calls with Negative Probesets” algorithm was used [59]. Differences in clinical parameters and cytogenetic analyses and between defined groups were investigated by exact Wilcoxon rank-sum test. Correlation was assessed using Pearson's correlation or Kendall's tau (for categorical variables) tests. The relationship between categorical variables was assessed using Fisher's exact test. Differential gene expression was assessed using empirical Bayes statistics in linear models for microarray data [60]. P-values were adjusted for multiple testing controlling the false discovery rate as defined by Benjamini and Hochberg [61]. All computations were performed using R 2.14.1 (http://www.r-project.org/), and Bioconductor 2.9 [62]. EFS and OS were investigated using Cox's proportional hazard model as previously published [53]. For EFS and OS, cut-offs were calculated as the mean cut-off from EFS and OS, respectively. Gene expression-based assessment of risk and proliferation as well as classifications of myeloma was performed as previously published [2]. Findings were confirmed using the same strategy on the independent group of 701 patients treated within the total therapy 2 and 3 protocols, respectively [63, 64]. Microarray data are available in the public databases under accession numbers E-TABM-1138 and GSE24080. An effect was considered as statistically significant if the *P*-value of its corresponding statistical test was not higher than 5 %.

### Transfections

U-266 cell were transfected with the pCMV survivin, the pCMV Mcl-1 plasmid [65] or a control plasmid (pCMV-Tag4A, Stratagene, La Jolla, CA) using the Neon Transfection System (Invitrogen, Carlsbad, CA).

### Cell-based Assays

Cell growth and Caspase 3/7 assays were conducted in 96-well plates according to the manufacturer's instructions using CellTiter-Glo™ and Caspase-Glo 3/7™ assays from Promega (Madison, WI). Cell proliferation was assessed by measuring [3H]-thymidine uptake, as described in prior studies [36].

### Isobologram analysis

For combination studies, data from CellTiter Glo™ assays were converted into values representing the fraction of growth affected (FA) in drug-treated versus untreated cells and analyzed using the CalcuSyn software program (Biosoft, Ferguson, MO) based on the Chou-Talalay method. A combination index (CI) < 0.9 indicates synergism, whereas 0.9 to 1.1 indicates additive effects.

### SDS-PAGE and Western Immunoblot

Cell lysis, immunoprecipitation, and Western blot analysis were performed as described previously [36]. Briefly, after washing with phosphate-buffered saline, cells were pelleted and resuspended in lysis buffer (Tris/HCl pH 8.0 50 nM; NaCl 150 mM; Nonidet P40 1% (v/v); EDTA 1mM; sodium deoxycholate 0.25% (w/v)) containing protease and phosphatase inhibitors (Complete Protease Inhibitor Cocktail Tablets and PhoSTOP Phosphatase Inhibitor Cocktail Tablets, Roche, Basel, Switzerland). Proteins were separated by sodium dodecyl sulfate polyacrylamide gel electrophoresis (SDS-PAGE) and afterwards blotted onto nitrocellulose membranes (GE Health Care, Buckinghamshire, UK). All membranes were blocked with 5% milk in TBST (Tris/HCl pH 7.4; NaCl 137 mM; KCl 2.7 mM; Tween 0.1% (v/v)) for one hour before incubation with the respective primary antibodies. The horseradish peroxidase conjugated secondary antibodies were purchased from Santa Cruz Biotechnology (Santa Cruz, CA) and the ECL-Kit from Thermo Fisher Scientific (Thermo Fisher Scientific, Bonn, Germany).

### Gene expression analysis

U-266 cells were treated with YM155 at concentrations of 4 nM and 50 nM for 6 and 12 hours as well as 36 hours (only 4nM). Control cells were cultivated with solvent only (DMSO, Merck, Darmstadt, Germany). Total RNA isolation using the RNeasy Kit (Qiagen, Hilden, Germany) was repeated independently and both biological replicates were used for microarray analysis using the Illumina chip HumanHT-12 v4 (Illumina, San Diego, CA). Data were analyzed using Ingenuity Pathway Analysis (Ingenuity Systems, Redwood City, CA). Only genes with a significant fold change (Benjamini-Hochberg adjusted p-value < 0.05) were included in the analysis.

### Indirect immunofluorescence staining

U-266 and INA-6 cells were seeded on cover slips precoated with poly-l-lysin. Indirect immunofluorescence staining was carried out as previously described [66]. Fluorescence images were captured and processed using a Zeiss Axiovert 200 M microscope and Axiovison Software (Zeiss, Göttingen, Germany).

## SUPPLEMENTARY MATERIAL FIGURES


