# Supplementary material for: Preclinical efficacy of sepantronium bromide (YM155) in multiple myeloma is conferred by down regulation of Mcl-1
Source: Oncotarget. 2014 Sep 26;5(21):10237–50. doi: 10.18632/oncotarget.2529 (PMC4279369; doi:10.18632/oncotarget.2529)
Supplement: Supplementary file 1 [file oncotarget-05-10237-s001.pdf]

Preclinical efficacy of sepantronium bromide (YM155) in multiple myeloma is conferred by down regulation of Mcl-1

Supplementary Material

A

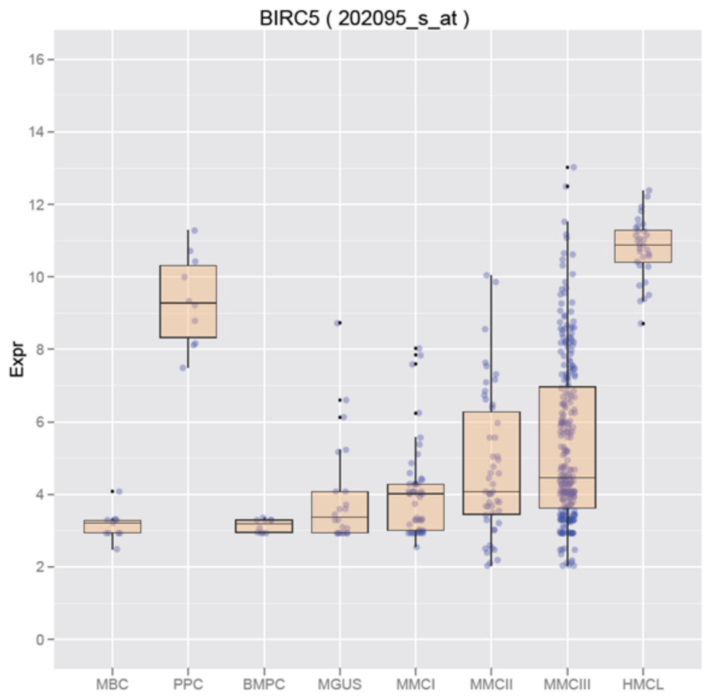

B

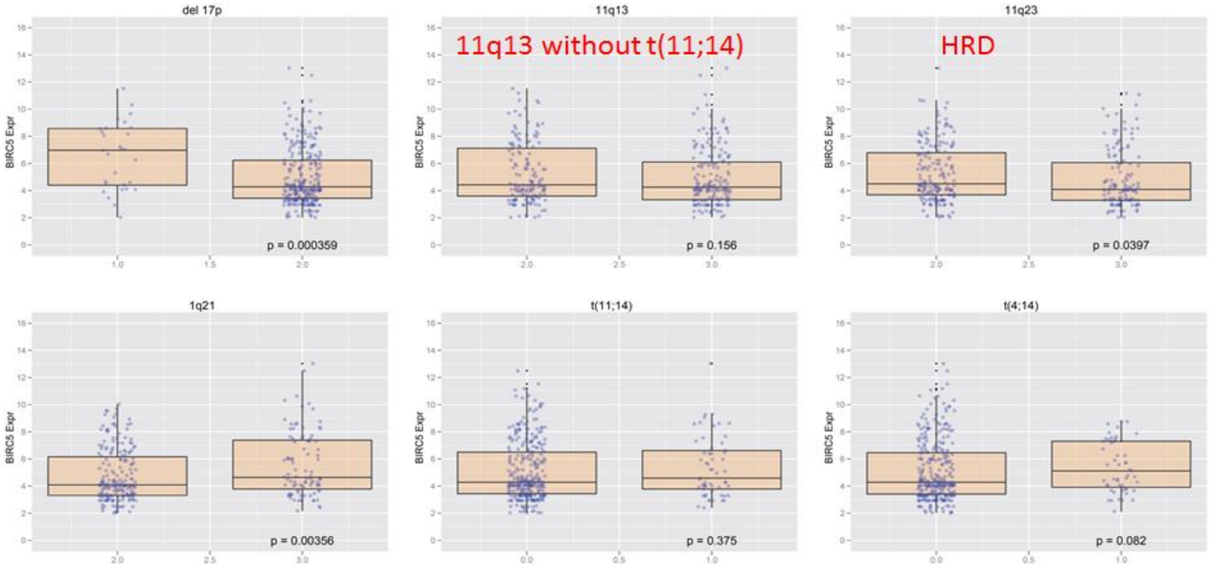

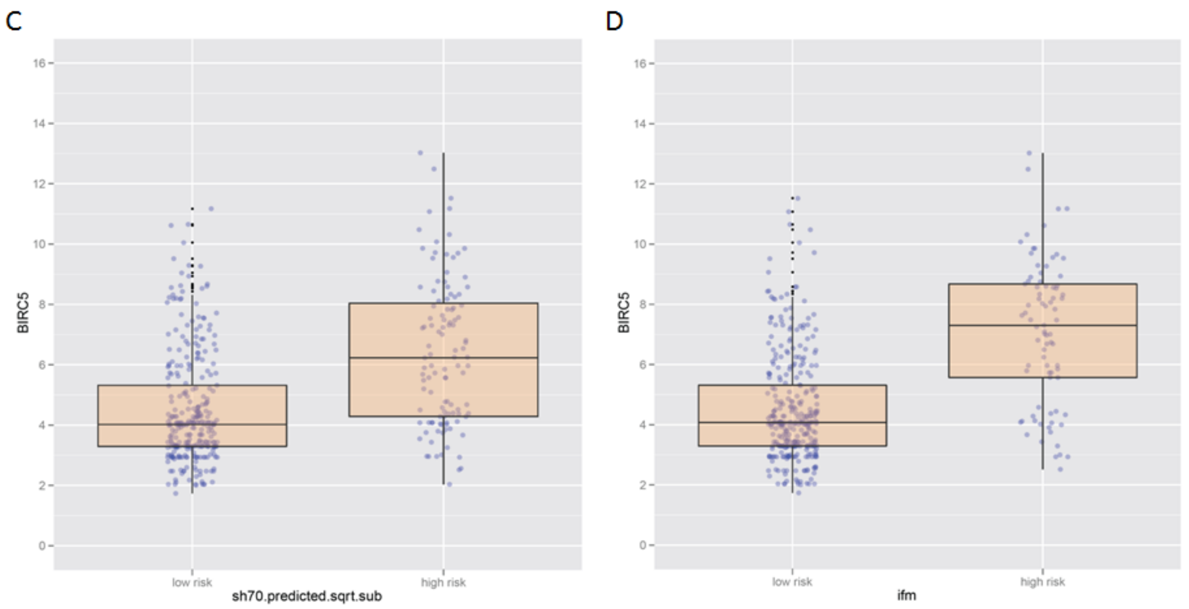

Suppl. Figure 1: Association of survivin gene expression with disease stage, cytogenetics, and gene expression based risk scores

(A) *BIRC5* levels as shown in corresponding figure 1A, subdividing MM samples according to the respective Durie-Salmon stages ($P < 0.001$). (B) Logarithmic scale of *BIRC5* expression in samples with or without the indicated cytogenetic aberrations. Numbers on the X-axis represent the frequency of the respective alleles or the presence/absence of a translocation

(C) *BIRC5* expression in low risk and high risk patients as assessed by the gene expression based risk scores UAMS 70 gene score and the IFM-score.

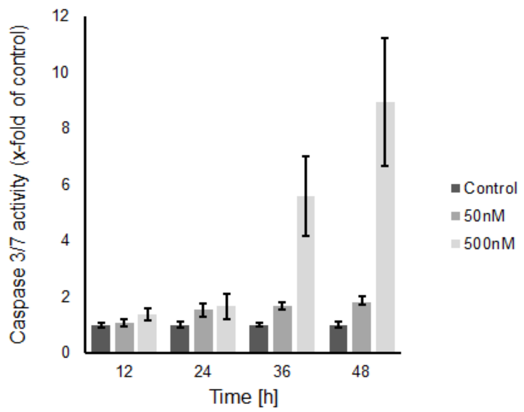

Suppl. Figure 2: Apoptosis induction in OPM-2 cells by YM155

Caspase 3/7 activity in OPM-2 cells treated with either 50nM or 500nM of YM155 compared to control cells after 12, 24, 36 and 48 hours.

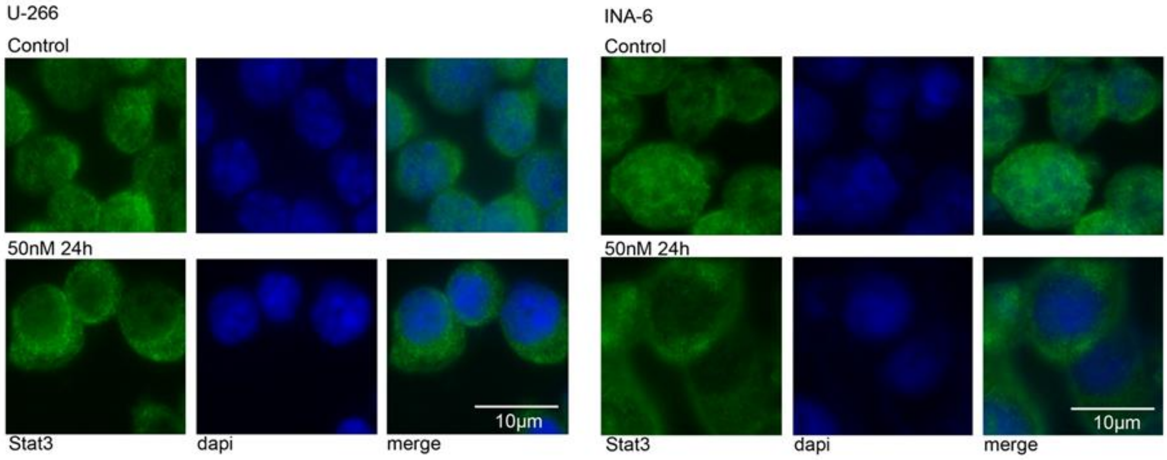

Suppl. Figure 3: Nuclear translocation of STAT3 under YM155 treatment

Active nuclear STAT3 (phospho-STAT3) levels in YM155 treated and control cells were investigated in a STAT3 immunofluorescence staining. Cells were fixed after 24 hours of incubation. DAPI was used to stain DNA.

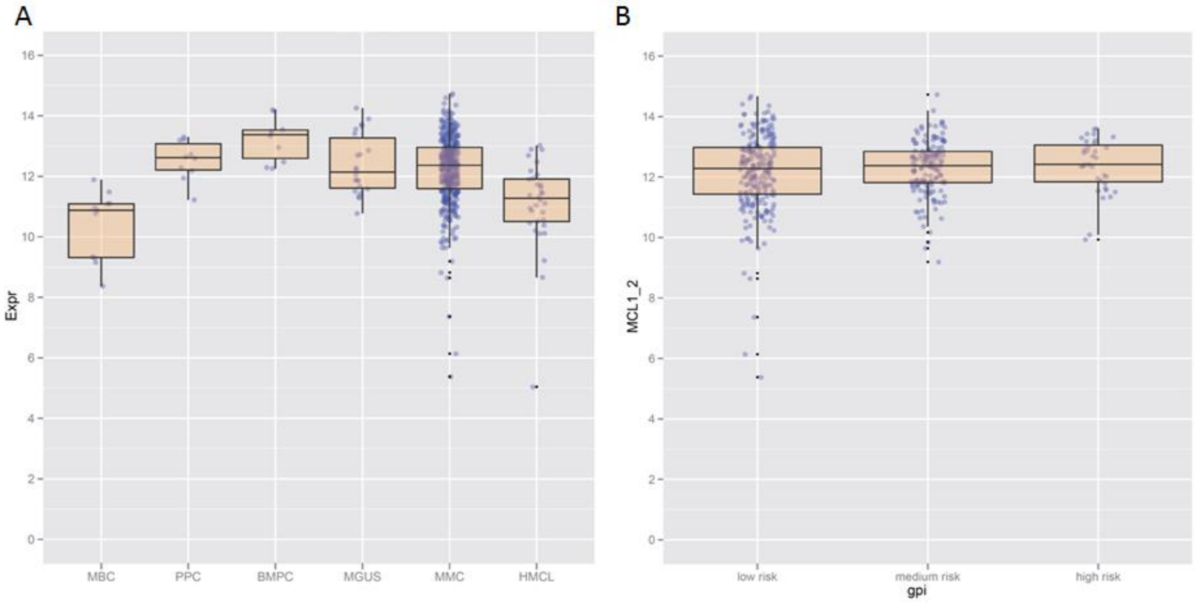

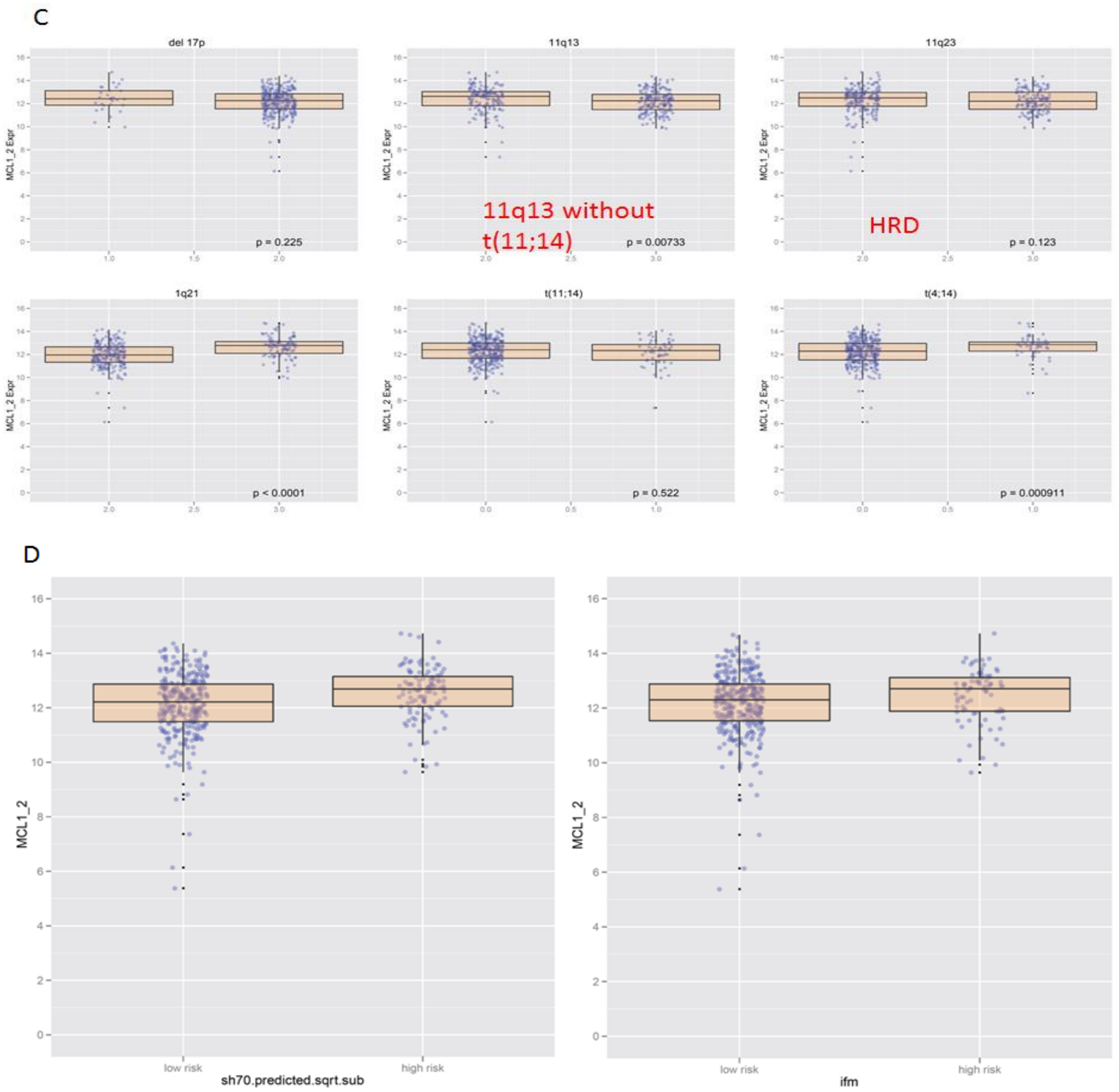

Suppl. Figure 4: *MCL1* expression in multiple myleoma and association with cytogenetics and gene expression based risk scores

(A) Logarithmic scale of *MCL1* expression in MBC (memory B-cells), PPC (polyclonal plasmablastic cells), BMPC (bone marrow plasma cells), MGUS, MMC (multiple myleoma cells) and HMCL (human myleoma cell lines). (B) Logarithmic scale of *MCL1* expression in patient samples with low, medium or high risk GPI. (C) *MCL1* expression in samples with or without the indicated cytogenetic aberrations. Numbers on the X-axis represent the frequency of the respective alleles or the presence/absence of a translocation. (D) *MCL1* expression in low risk and high risk patients as assessed by the gene expression based risk scores UAMS 70 gene score and the IFM-score.

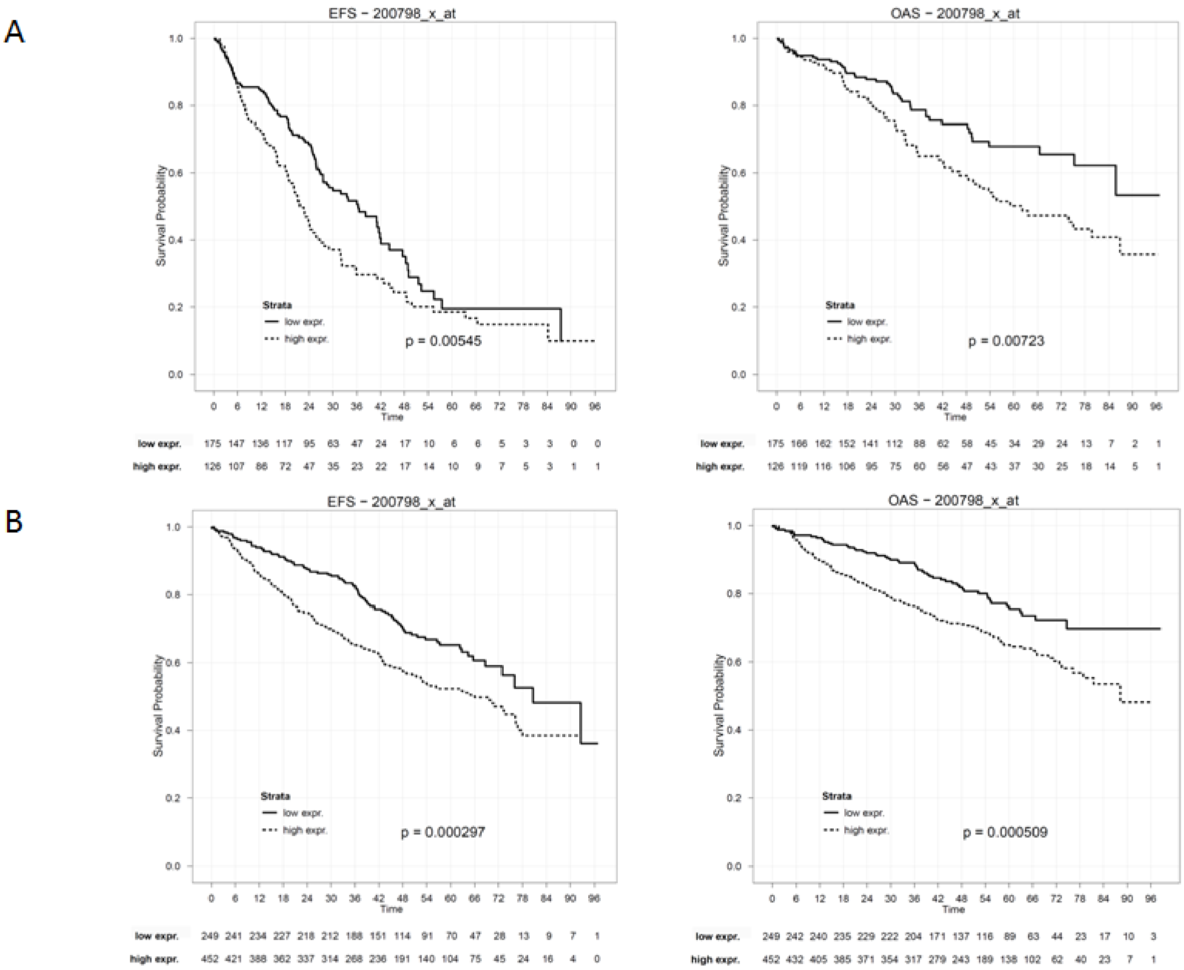

Suppl. Figure 5: *MCL1* expression and survival in multiple myeloma

Upper panel: Event-free and overall survival in 301 patients undergoing high-dose chemotherapy at our center in Heidelberg with low or high *MCL1* expression, respectively.

Lower panel: UAMS Arkansas data: 701 patients treated within the total therapy 2 and 3 protocol.
